# Construction and validation of a two-gene signature based on SUMOylation regulatory genes in non-small cell lung cancer patients

**DOI:** 10.1186/s12885-022-09575-4

**Published:** 2022-05-23

**Authors:** Hongxu Sheng, Zhexue Hao, Linhai Zhu, Yuan Zeng, Jianxing He

**Affiliations:** 1 Department of Thoracic Surgery and Oncology, The First Affiliated Hospital of Guangzhou Medical University, China National Center for Respiratory Medicine, China State Key Laboratory of Respiratory Disease & National Clinical Research Center for Respiratory Disease, Guangzhou, China; 2 Department of Thoracic Surgery, The First Affiliated Hospital, School of Medicine, Zhejiang University, Zhejiang, Hangzhou China

**Keywords:** SUMOylation, Gene signature, Overall survival, Nomogram, Non-small cell lung cancer

## Abstract

**Background:**

Post-translational modification plays an important role in the occurrence and development of various tumors. However, few researches were focusing on the SUMOylation regulatory genes as tumor biomarkers to predict the survival for specific patients. Here, we constructed and validated a two-gene signature to predict the overall survival (OS) of non-small cell lung cancer (NSCLC) patients.

**Methods:**

The datasets analyzed in this study were downloaded from TCGA and GEO databases. The least absolute shrinkage and selection operator (LASSO) Cox regression was used to construct the two-gene signature. Gene set enrichment analysis (GSEA) and Gene Ontology (GO) was used to identify hub pathways associated with risk genes. The CCK-8 assay, cell cycle analysis, and transwell assay was used to validate the function of risk genes in NSCLC cell lines.

**Results:**

Firstly, most of the SUMOylation regulatory genes were highly expressed in various tumors through the R package ‘limma’ in the TCGA database. Secondly, our study found that the two gene signature constructed by LASSO regression analysis, as an independent prognostic factor, could predict the OS in both the TCGA training cohort and GEO validation cohorts (GSE68465, GSE37745, and GSE30219). Furthermore, functional enrichment analysis suggests that high-risk patients defined by the risk score system were associated with the malignant phenomenon, such as DNA replication, cell cycle regulation, p53 signaling pathway. Finally, the results of the CCK-8 assay, cell cycle analysis, and transwell assay demonstrated that the two risk genes, *SAE1* and *UBA2*, could promote proliferation and migration in non-small cell lung cancer cells.

**Conclusions:**

The two-gene signature constructed in our study could predict the OS and may provide valuable clinical guidance for the treatment of NSCLC patients.

**Supplementary Information:**

The online version contains supplementary material available at 10.1186/s12885-022-09575-4.

## Background

Lung cancer is considered the leading cause of cancer mortality, worldwide, with an approximate 1.8 million deaths in 2020 [1]. Due to recent advances in early detection and treatment, the survival rate of lung cancer continues to increase worldwide. However, there are still patients who are in an advanced or locally advanced stages at their initially diagnosis, especially those who lives in the less economically developed areas [2], and for these patients the 5-year overall survival (OS) rate remains very low [3]. Therefore, the identification of biomarkers and risk factors that will help to develop new drugs able to improve OS is still an important and valuable task.

Protein post-translational modifications (PTMs) are chemical modifications achieved by the covalent addition of functional proteins, proteolytic cleavage of regulatory subunits, or degradation of entire proteins. These modifications include phosphorylation, glycosylation, ubiquitination, SUMOylation, acetylation, and methylation, which are involved in almost all aspects of normal cell biology and pathogenesis [4–8]. Recently studies showed that proteins modified by the SUMO family are commonly dysregulated in various tumors and are involved in several biological processes, such as cell cycle [9], proliferation [10], and metastasis [11, 12]. It has been suggested that the SUMOylation modification plays an important role in the occurrence and development of the disease, and may contribute to the development of new drug targets to improve the clinical treatment of NSCLC in the future.

In this study, we used a cohort extracted from The Cancer Genome Atlas (TCGA)-lung adenocarcinoma (LUAD) dataset as the training set and included the relevant E1, E2, E3, and deSUMOylation enzymes involved in the SUMO modification process to construct a risk model. A set of risk assessment models was constructed through the least absolute shrinkage and selection operator (LASSO) Cox analysis. After survival analysis, it was found that our risk assessment model could predict the OS of LUAD patients, which was verified using GEO datasets. Next, to evaluate the applicability of the model, we further expanded the sample type and verified the validity of the model in the NSCLC patients of GEO datasets. We found that our risk prediction model could also distinguish OS of NSCLC patients. Finally, the role of risk genes, *SAE1* and *UBA2*, was verified in NSCLC cell lines in cell proliferation, effects on the cell cycle, and migration. We believe that the risk model constructed in this study would be valuable in selecting patients at a high risk of relapse or to provide clinical guidance for the treatment of patients with NSCLC.

## Materials and methods

### Datasets

The processed mRNA expression profiles and clinical data of LUAD samples (*n* = 477) was download from the TCGA database (https://portal.gdc.cancer.gov) through R packages “TCGAbiolinks” [13]. The processed expression matrix and clinical data of validation cohorts (GSE68465, GSE37745, and GSE30219) were acquired from the website of GEO database (https://www.ncbi.nlm.nih.gov/geo/). Log2 (FPKM + 1) transformed normalized values were used for analysis. As all data analyzed in this study were openly acquired from TCGA and GEO databases, ethics approval was not obtained and the need for informed consent was waived.

### Data processing

To use the model derived from the TCGA training cohort in the validation cohorts of GEO, we corrected the expression profile data of TCGA and GEO using the R package “limma” and “sva” for verification. The data used in this study contain corresponding survival information, and samples with survival less than 30 days or incomplete clinical information were removed. Due to the tissue expression specificity of *SUMO-4*, there were some cases (42 of 595) with missing gene expression data, and for these genes we used median expression values to replace the missing data.

### Selection of SUMOylation regulatory genes

As a post-translational modification, the process of SUMOylation is similar to that of ubiquitination, and requires E1-activating enzymes (*SAE1* and *UBA2*), an E2-conjugating enzyme (*UBE2I*), and E3 ligases (*PIAS1, PIAS2, PIAS3, PIAS4, RanBP2,* and *CBX4*). SUMO-specific peptidase enzymes (*SENP1, SENP2, SENP3, SENP5, SENP6,* and *SENP7*) could catalyze the deSUMOylation, and we also included SUMOs genes in this study. Furthermore, SUMO may covalently modify the PML protein [14], and the modified PML is localized in a nucleosome called polyprotein, which becomes nuclear bodies (PML-NBs) [15]. PML-NBs can be used as a scaffold and is modified by SUMO to further recruit other proteins, to regulate protein activity and function [14, 16–18]. Therefore, PML was also included in this study along with SUMOs, E1, E2, E3 and, SENPs.

### Construction of a risk scoring system

The LASSO Cox analysis is a widely used dimensionality reduction analysis method [19]. By regressing and penalizing all variables, the coefficients of relatively unimportant variables became zero and were excluded from the model. Subsequently, the independent variables shown to has a greater impact on the dependent variable were used to calculate the corresponding regression coefficient, which is an optimal approach to construct a signature if there are numerous correlated covariates. The LASSO Cox regression analysis was used in the training set to construct a risk scoring system (RSS) by a linear combination of the risk genes defined by their calculated regression coefficients. Furthermore, the prognostic prediction efficiency of this RSS was further validated in the GEO cohorts.

### Gene set enrichment analysis

Gene set enrichment analysis (GSEA) was applied in the TCGA training cohort to explore the critical pathways closely associated with high-risk groups divided by risk genes through the R package “ClusterProfiler” [20]. A *P*-value < 0.05 was considered statistically significant.

### Oncomine database

The expression of SUMOylation regulatory genes across cancer types was analyzed in Oncomine which is a public access online database (https://www.oncomine.org) [21].

### Cell lines and cell culture

The A549 and H838 cell lines were cultured in RPMI-1640 medium (Corning, Inc.) supplemented with 10% fetal bovine serum (FBS, Corning, Inc.), 100 U/mL penicillin, and 100 μg/mL streptomycin at 37 °C in an incubator with 5% CO_2_. Both cell lines were purchased from The Cell Bank of Type Culture Collection of the Chinese Academy of Sciences.

### Plasmids transfection

*SAE1* and *UBA2* plasmids were purchased from Vigenebio (Shandong, China). The transfection reagent, Lipofectamine 3000 (Invitrogen, USA) was used to perform the plasmids transfection according to the manufacturer’s instructions. After 60 h of transfection, cells were collected for detecting the transfection efficiency through western blot.

### Western blot analysis

In brief, after transfection with indicated plasmids for 60 h, cells were collected and lysis in RIPA containing protease inhibitor (EMD Millipore, USA). Following quantitation through BCA assay, proteins were separated by a 10% SDS-PAGE gel. Subsequently, the membrane was blocked for 1 h at room temperature and incubated with relevant primary SAE1 (cat. no. ab185552; 1:10000; Abcam), UBA2 (cat. no. ab185955; 1:1000; Abcam), and SUMO1 (cat. no. 4930; 1:1000; Cell Signaling Technology). Protein were incubated with horseradish peroxidase (HRP)-conjugated anti-rabbit IgG (cat. no. 7074; 1:2000; Cell Signaling Technology) and detected by enhanced chemiluminescence assay.

### Cell proliferation assay

Cell proliferation was determined using a CCK-8 assay (Biosharp life science, Anhui, China). After transfection of *SAE1* and *UBA2* plasmids, cells were seed into 96-well plates (1.5 × 10^3^ cells/well) and 10 μL CCK-8 solution was added into each well at the indicated time, incubated for 90 min. The optical density (OD) of the lysate was measured at 450 nm using a microplate spectrophotometer. Each experimental group was repeated a total of five times.

### Transwell assay

Cell migration ability was measured by Transwell assay. After transfection of *SAE1* and *UBA2* plasmids simultaneously, approximately 5 × 10^4^ A549 and H838 cells were resuspended with serum-free medium and seeded into the upper chambers. Subsequently, 900 μL medium supplemented with 20% FBS was added to the lower chamber. After a 24-h co-culture, the cells in the upper chamber were removed and the membrane was fixed in 4% paraformaldehyde for 15 min. Finally, the membrane was stained with 0.1% crystal violet and migrated cells were counted under an inverted microscope.

### Cell cycle analysis

After transfection with *SAE1* and *UBA2* plasmids, A549 and H838 cells were collected and fixed at − 20 °C by 70% ethanol overnight. Following centrifugation at 500×g for 5 min at room temperature, cells were washed three times with PBS. Then 1 mL DNA staining solution Propidium iodide (PI) was added into the tube and the cell suspension was incubated for 30 min at room temperature and protected from light. A flow cytometer was used to examine the cell cycle distribution and FlowJo v10.4 software was used for analysis.

### Statistical analysis

The Wilcox test was used to evaluate the differential expression of the 20 SUMOylation regulatory genes. Survival curves were plotted by the R package “Survival” and “Survminer”. A nomogram was constructed using the R package “rms”. Univariate and multivariate Cox regression analyses were used to identify potential clinicopathological characteristics as independent prognostic factors. R software (version 3.5.1) was used to carry out the statistics and GraphPad Prism 8 software was used to perform unpaired Student’s t-tests to analyze differences between two groups. A *P*-value < 0.05 was considered to be a significant difference.

## Results

### Clinical relevance of SUMOylation regulatory genes across cancer types

**Fig. 1:**
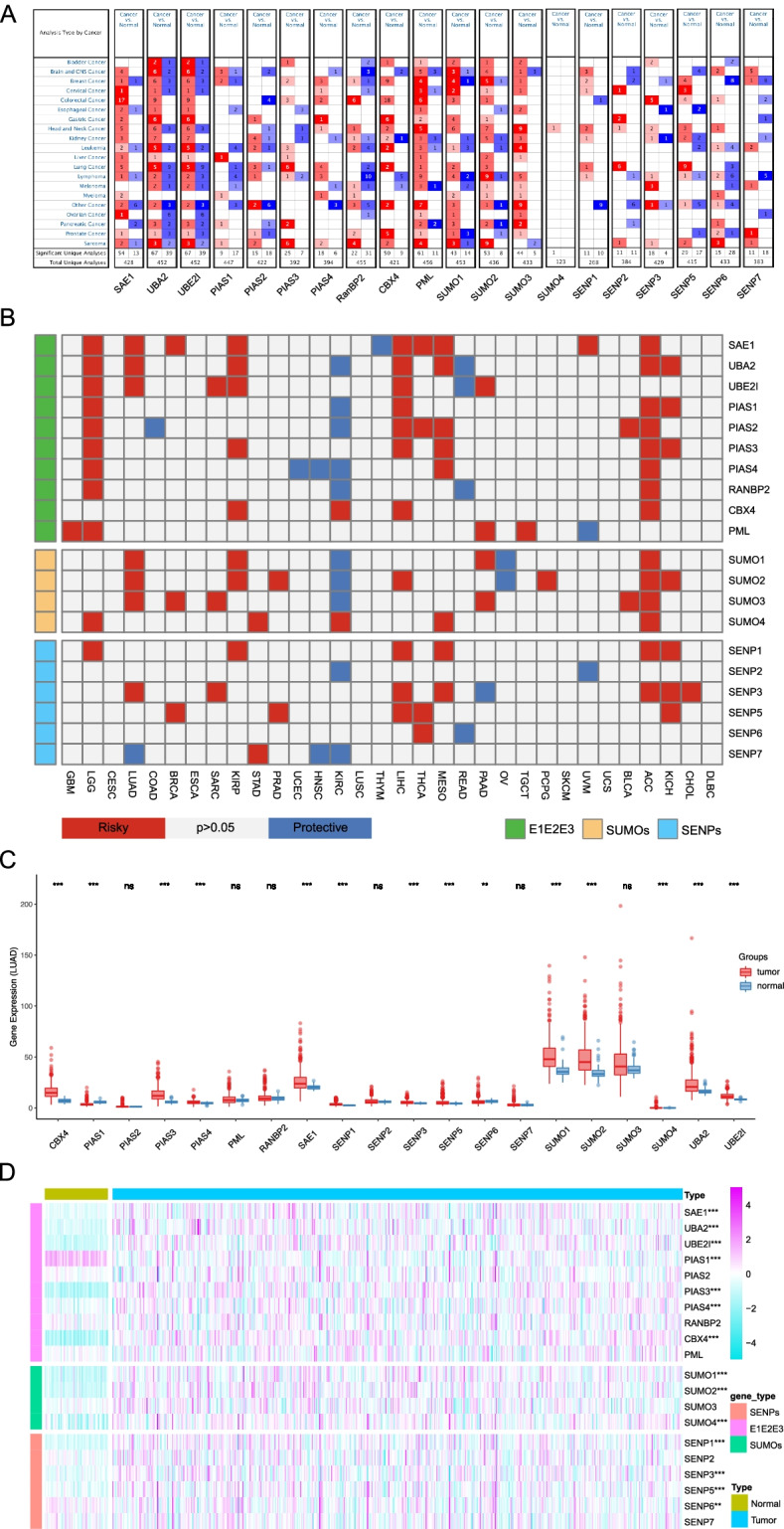
The expression of SUMOylation regulatory genes across cancer types. **A** Summary expression of the 20 SUMOylation regulatory genes in various tumors versus corresponding normal tissue from the Oncomine database. **B** Summary of the correlation between expression of SUMOylation regulatory genes and patient survival. Red represents worse survival associated with a higher expression of SUMOylation regulatory genes, and blue represents the better survival. **C** Box plot of the expression of SUMOylation regulatory genes in TCGA-LUAD. (D) The expression heatmap of SUMOylation regulatory genes in TCGA-LUAD. *P < 0.05, **P < 0.01, and ***P < 0.001

To better understand the roles of SUMOylation regulatory genes in the development of tumors, we evaluated the expression of those genes in various tumor tissues compared to that of corresponding normal tissues via the Oncomine database. As shown in Fig. 1A, SUMOylation regulatory genes were highly expressed in multi-tumor tissues. Next, we explored the correlation between the expression of SUMOylation regulatory genes and patient survival across cancer types. As shown in Fig. 1B, a higher expression of most genes was associated with worse survival, indicating that SUMOylation may have some effects on tumor development. Finally, we examined the expression of twenty genes in LUAD. Compared to normal tissue samples, *SAE1, UBA2, UBE2I, PIAS1, PIAS3, PIAS4, CBX4, SUMO1, SUMO2, SUMO4, SENP1, SENP3, SENP5,* and *SENP6* were significantly up-regulated in LUAD tissue samples (Fig. 1C, D).

### Construction and validation of the risk scoring system

**Fig. 2:**
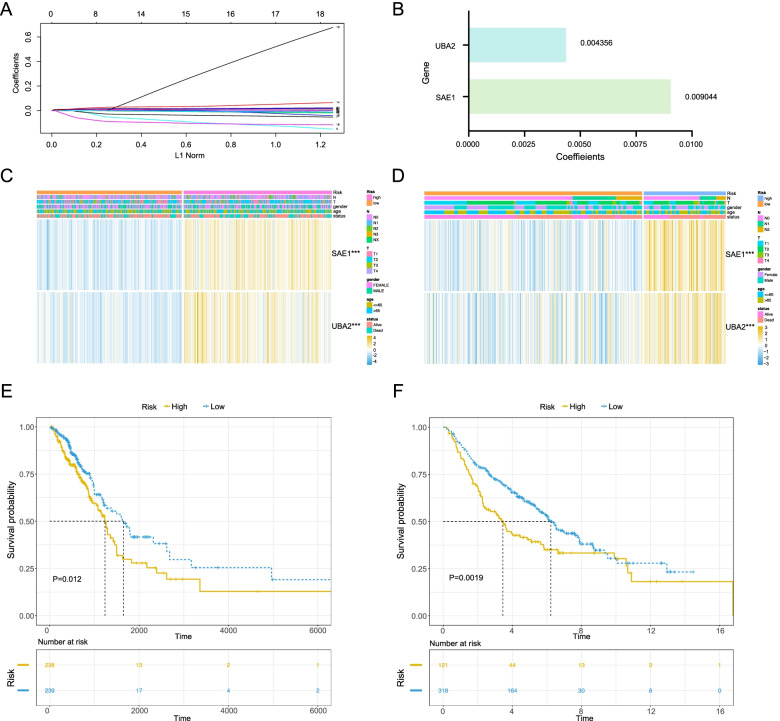
Development and validation of the prognostic model based on SUMOylation regulatory genes. **A, B** The cv-fit and coefficient profiles of the SUMOylation related genes calculated by LASSO regression analysis. **C, D** Heatmap of the expression and clinicopathological characteristics of *SAE1* and *UBA2* in high-risk and low-risk groups in the TCGA training cohort and GEO validation cohort. **E, F** Survival curve of risk genes in TCGA and GEO cohorts, respectively

To construct the risk scoring system, we used the LASSO Cox regression in the training cohort. Furthermore, we corrected for the expression of SUMOylation regulatory genes in TCGA-LUAD training cohort and GEO validation cohorts via R software to better examine the risk scoring system. Intersection of the gene expression in training cohort and validation cohort were subjected to further analysis. As shown in Fig. 2A and Supplemental Fig. 1A, a gene signature including two genes, *SAE1* and *UBA2*, was established after the LASSO Cox regression. Subsequently, we constructed a risk score by linear combination of each sample (risk score = 0.009044*SAE1 + 0.004356*UBA2) (Fig. 2B). Using the median value of risk score of all samples as the cutoff, the TCGA-LUAD training cohort could be classified into two groups: high- and low-risk groups. Next, we explored the expression levels of the two risk genes in high- and low-risk groups. The results showed that the two genes, *SAE1* and *UBA2*, were both highly expressed in the high-risk group (Fig. 2C). The event and risk score distribution of the training cohort was shown in Supplemental Fig. 1B, D. Moreover, patients in the low-risk group had an obviously longer OS than those in the high-risk group (*P* = 0.012) (Fig. 2E). To examine the robustness of the two-gene risk signature, the GSE68465 dataset was used to validate using the constructed risk score system. Similar to the results in the TCGA training cohort, compared with the low-risk group, the expression of *SAE1* and *UBA2* was significantly higher in the high-risk group (Fig. 2D). The event and risk score distribution of the validation cohort is displayed in Supplemental Fig. 1C, E. Meanwhile, the survival curve indicated that the OS of patients in the low-risk group was significantly higher than that in the high-risk group (*P* = 0.0019) (Fig. 2F**)**.

### Development of a nomogram for predicting overall survival in LUAD

**Fig. 3:**
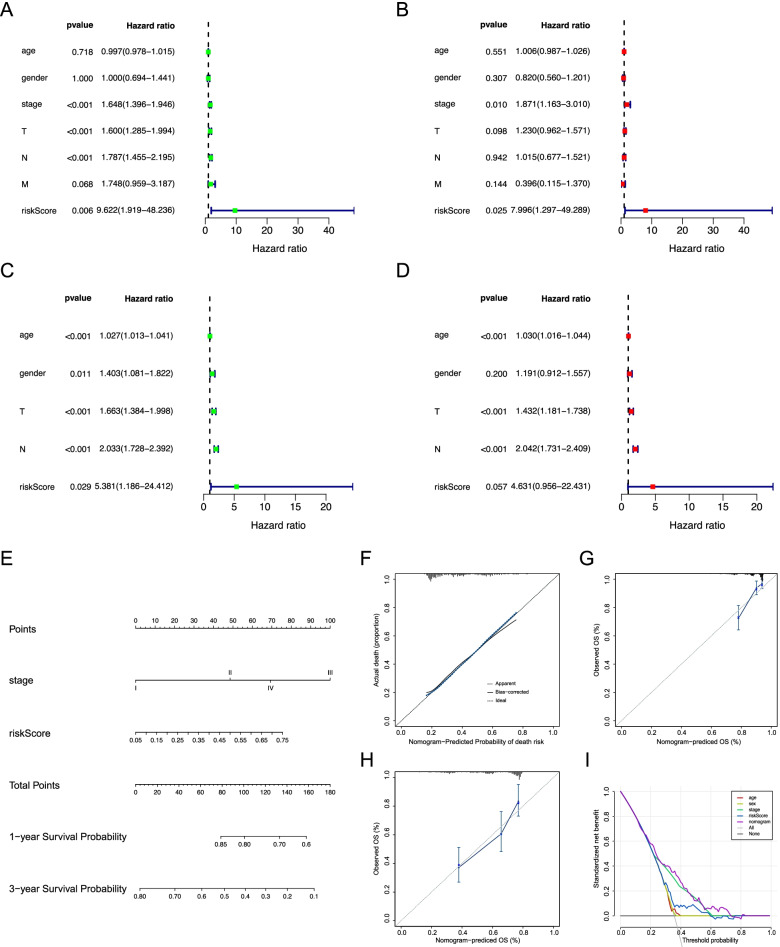
Construction and validation of the prognostic model. **A, B** Forest plot of univariate and multivariate Cox analysis in TCGA training cohort. **C, D** Forest plot of univariate and multivariate Cox analysis in the GSE68465 validation cohort. **E** Nomogram model for predicting the probability of 1-, and 3-year OS in the TCGA cohort. **F-H** Calibrations plots of the nomogram predicting 1-, and 3-year OS in the TCGA cohort. **I** Decision curve analysis of the nomogram in TCGA cohort compared with other models

Next, we carried out univariate and multivariate Cox regression analysis to verify whether the risk score system could act as an independent prognostic factor for predicting OS. The results showed that the pathological stage, T stage (primary tumor), N stage (regional lymph node), and the risk score was significantly associated with OS in univariate analysis (Fig. 3A) while only the pathological stage and risk score were still significantly associated with OS in multivariate Cox regression analysis (Fig. 3B). Subsequently, we further examined whether the risk score was an independent factor to predict OS in the GEO validation cohort. As shown in Fig. 3C, D (Supplemental Fig. 2A, B), similar with results for the TCGA training cohort, the univariate and multivariate Cox regression analysis in the GEO validation cohort confirmed that the constructed risk score also could be an independent prognostic factor, while in multivariate Cox regression analysis the risk score was weakly statistically significant (*P* = 0.057). In order to accurately predict the prognosis of LUAD patients, we then construct a nomogram incorporating those factors that are related to survival in the Cox regression analysis. The C-index value for the nomogram was 0.733 (CI, 0.681-0.787) (Fig. 3E). Subsequently, the 1-, and 3-year OS were used to evaluate the predictive accuracy of the nomogram (Fig. 3F-H**, **Supplemental Fig. 2C-E). Finally, to evaluate the clinical utility of the nomogram, we applied the decision curve analysis (DCA) to estimate the benefits of the different models (Fig. 3I). Furthermore, the AUC also was used to validate the clinical utility of the nomogram in the GSE30219 dataset. The results showed that the AUC value of the prognostic model for 5-year survival of patients was 0.687 (Supplemental Fig. 2F).

### Verification of OS prediction of risk scoring system in NSCLC

**Fig. 4:**
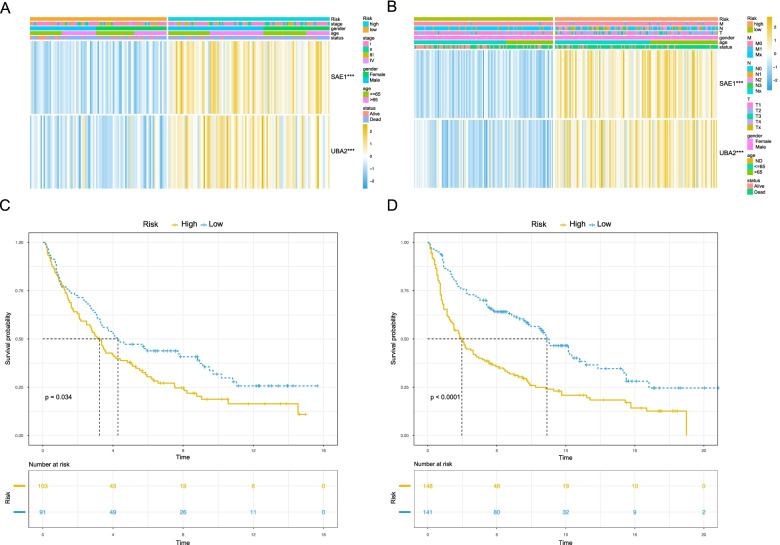
The prognostic model predicts the overall survival of NSCLC patients. **A, B** Heatmap of the expression and clinicopathological characteristics of *SAE1* and *UBA2* in GSE37745 and GSE30219 datasets, ***P < 0.001. **C, D** Survival curves of *SAE1* and *UBA2* in the GSE37745 (*P =* 0.034) and GSE30219 (*P <* 0.0001) cohorts, respectively

To verify the clinical application of this risk scoring system, we further validated the model using two NSCLC cohorts from the GEO database: GSE37745 (*n* = 196) and GSE30219 (*n* = 307). In each cohort, patients were classified into high- and low-risk groups based on the cutoff obtained from the TCGA training cohort. The generated heatmap showed that the expression of *SAE1* and *UBA2* was highly expressed in the high-risk group compared with the low-risk group (Fig. 4A, B). Next, the risk score and event distribution in the two GEO validation cohorts were compared, as shown in Supplemental Fig. **3**. Finally, we obtained similar results as in the TCGA training cohort in terms of survival, patients in the high-risk group had a significantly shorter OS in both GSE37745 (Fig. 4C) and GSE30219 (Fig. 4D) datasets, which indicated that the model could be applied to NSCLC patients. Taken together, the results suggested that the prognosis model we constructed in this study could also predict the OS of NSCLC patients.

### Gene ontology and gene set enrichment analysis

**Fig. 5:**
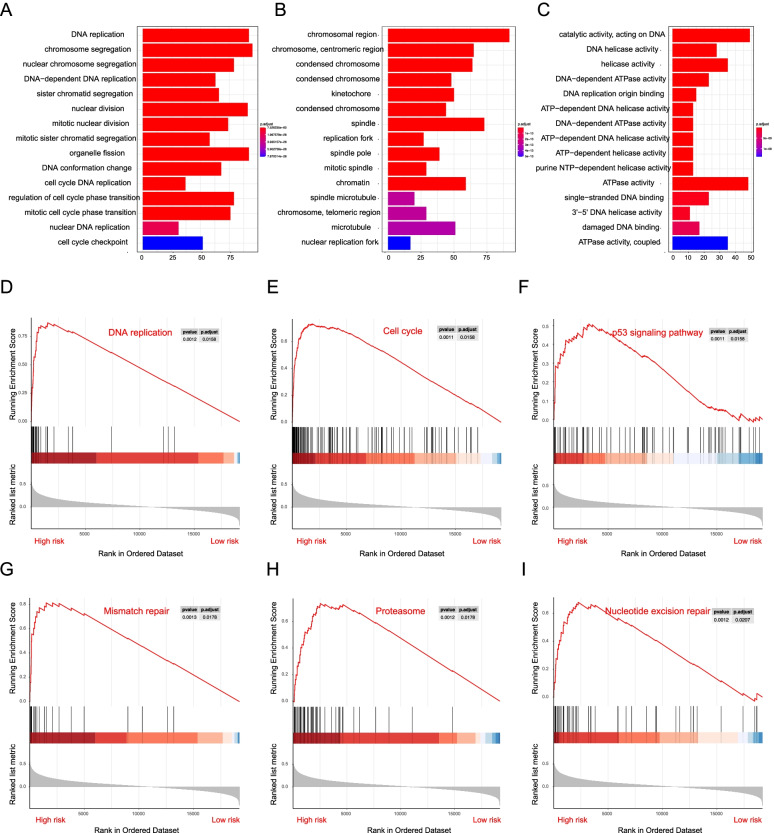
The association between high-risk group patients and cancer-related pathway. **A-C** the top enriched biological processes, cellular components, and molecular functions analysis of high- versus low-risk groups. **D-I** The cancer-related pathways enriched in high-risk group patients

Based on the prognostic function of risk genes, we further investigated the Gene Ontology (GO) between risk groups using the R package “ClusterProfiler”. Enrichment results showed that the high-risk group was mainly associated with altered cell cycle, DNA replication, and chromosome segregation, effects which are mostly related to tumorigenesis (Fig. 5A-C). Subsequently, GSEA was then performed to compare the risk groups. The results showed that the malignant hallmarks of cancer, including DNA replication (NES = 2.66, *P* = 0.0012), cell cycle changes (NES = 2.787, *P* = 0.0011), the *p53* signaling pathway (NES = 1.922, *P =* 0.0011), mismatch repair (NES = 2.319, *P =* 0.0013), the proteasome (NES = 2.515, *P =* 0.0012), and nucleotide excision repair (NES = 2.272, *P =* 0.0012) were significantly correlated with the high-risk group (Fig. 5D-I), which suggested that the high-risk score may contribute to the development of tumor, cause that GSEA phenomenon were closely associated with tumor malignancy.

### Risk genes promote proliferation and migration in NSCLC cell lines

**Fig. 6:**
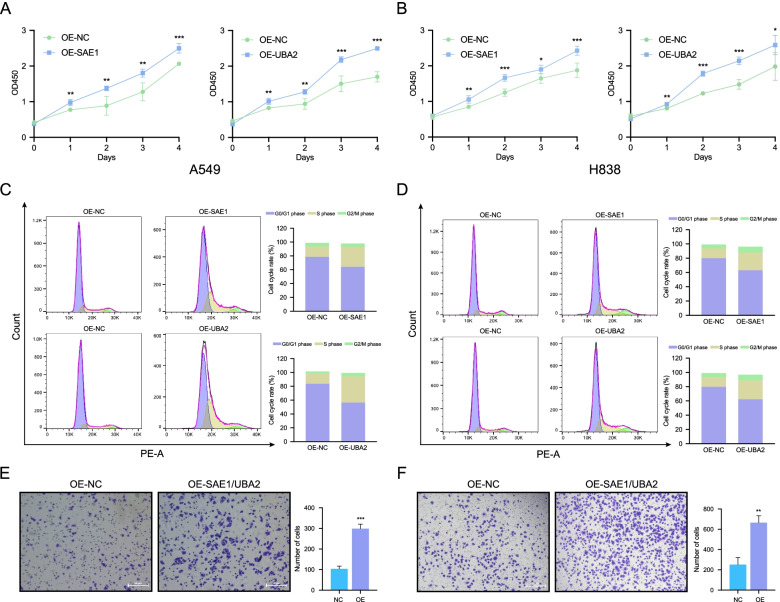
*SAE1* and *UBA2* promote the proliferation and migration in NSCLC cells. **A, B** The CCK-8 assay of A549 and H838 cells transfected with *SAE1, UBA2,* or negative control plasmids. **C, D** Cell cycle analysis by flow cytometry of A549 and H838 cells transfected with *SAE1, UBA2*, or negative control plasmids. **E, F** Transwell migration assay results from A549 and H838 cells co-transfected with *SAE1* and *UBA2*, or negative control plasmids. *P < 0.05, **P < 0.01, and ***P < 0.001

Through the above analysis, we identified potential roles of risk genes in the development of NSCLC. In order to validate the conclusion, we further explore the effects of the two risk genes, *SAE1* and *UBA2*, in vitro. Firstly, cell proliferation was tested using the CCK-8 assay. As shown in Fig. 6A **and** Fig. 6B, over-expression of *SAE1* and *UBA2* could promote the proliferation of A549 and H838 cells. Subsequently, cell cycle staining was performed to evaluate the cell cycle distribution in both cell lines. As shown in Fig. 6C and Fig. 6D, after transfection of *SAE1* and *UBA2* plasmids, the cell cycle transited from G1 to S phase in both A549 and H838 cells, indicating cell proliferation was accelerated by the two risk genes. Finally, we evaluated the impact of *SAE1* and *UBA2* on the potential migration of NSCLC cells using Transwell migration assays. Results shown that over-expression of *SAE1* and *UBA2* could significantly enhance the migration of the NSCLC cells (Fig. 6E and Fig. 6F).

## Discussion

Small ubiquitin-related modifiers 1, 2, 3, and 4 (*SUMO-1, − 2, − 3,* and *− 4*) are members of the ubiquitin-like protein family having a molecular weight of 10 kDa [22]. Similar to ubiquitin, the SUMO family can target different proteins with multiple biological functions [23]. Ubiquitin mainly regulates the degradation of its target protein, while SUMO proteins may couple multiple proteins to perform biological function [24–26].

In the present study, we attempted to develop and validate a novel risk score for predicting the OS in LUAD patients based on the expression of SUMOylation regulatory genes. Firstly, to explore the effects of regulatory genes in the occurrence and development of tumors, we verified the differential expression of 20 SUMOylation regulatory genes in the Oncomine and TCGA databases. The results showed that compared with the corresponding normal tissues, most of the 20 genes were highly expressed in various tumors, suggesting that they may have some effects on the occurrence and development of tumors. Subsequently, we constructed a two-gene signature including *SAE1* and *UBA2* using Lasso Cox regression analysis to predict the OS in LUAD patients. A significant distinction was observed between low- and high-risk patients in both the training cohort (TCGA-LUAD) and the validation cohort (GSE68465). Interestingly, we expanded the scope of application in the validation cohorts and found that this risk score system could also distinguish the OS of patients in NSCLC cohorts (GSE37745, GSE30219), indicating the broad application of this gene signature in prognosis prediction. Moreover, we incorporated age, sex, stage, TNM, and the risk score into univariable and multivariable Cox regression analyses. The results showed that the risk score was a prognostic factor in both the training cohort and validation cohorts, though it remained a weak significance in the multivariate Cox analysis of the GEO validation dataset (GSE68465, *P* = 0.057). As E1 activating enzymes, *SAE1* and *UBA2* are involved in various of tumors [11, 27]. It has been reported that *SAE1* can mediate the progression of human glioma by activating the AKT signaling pathway through SUMOylation [28]. *SAE1* is also closely related to the development of hepatocellular carcinoma, and is helpful for its diagnosis and prognosis [29]. In addition, *UBA2* has been reported to promote the progression of colon cancer, liver cancer, breast cancer, and other tumors [30–32]. In terms of the potential mechanisms, GSEA enrichment results showed that the malignant hallmarks of cancer, including disturbances in DNA replication, the cell cycle, the *p53* signaling pathway, mismatch repair, proteasome, and nucleotide excision repair, had significant correlations with the high-risk group. Li et al. found that SUMOylation-related regulatory molecules could be used as diagnostic markers for glioma and participates in its carcinogenesis. Functional enrichment analysis found that it is closely related to the cell cycle and DNA replication [33]. In our study, the findings of the functional enrichment analysis of SUMOylation regulatory molecules in NSCLC were essentially the same as those reported previously. In addition, Liu et al. reported that the SENP1 was a risk factor of NSCLC and could contribute to chemoradiotherapy resistance [34], indicating that SUMOylation regulatory genes may have an important role in various tumors. Besides, Ginkgolic acid, as a botanical drug, could inhibit the interaction of E1 enzymes and SUMO protein, which has been found its anti-tumor effects in ovarian cancer [35], endometrial cancer [36], gastric cancer [37], liver cancer [38], and lung cancer [39]. Consistent with the previous studies, our results of in vitro experiments also confirmed that targeting the E1-SUMO intermediate may have a potential anti-tumor effect.

In addition, Wu et al. analyzed the role of SUMOylation regulatory molecules in pan-cancer types and found that SUMOylation regulatory molecules presented extensive genetic variation and expression changes and may provide valuable reference for clinical diagnosis and treatment. Their research demonstrates that *SENP1*, *SENP7*, *SAE1*, and *TRIM27* could be used as risk genes to predict OS in LUAD [40]. In our study, we also found that *SAE1* could be considered as a risk gene. Different from previously published studies, the *UBA2* risk gene identified in our study may have the same importance as *SAE1* in the OS prediction. Furthermore, our risk model has a wider range of applications, as it can also predict the OS in NSCLC.

Despite the interesting findings in our study, there are still some limitations. Firstly, to make the model have certain applicability, only the common clinical information in TCGA training cohorts and GEO validation cohorts was included in this study. Thus, the lifestyle, mutation features, or other information were not included. Secondly, compared with the tumor stage, the nomogram we constructed had a certain discriminatory advantage, but this advantage was not as marked in the DCA curve analysis. Finally, SUMOylation is closely associated with immune function. In the future, we will further evaluate the relationship between our risk gene signature and immune cell infiltration in NSCLC disease.

In conclusion, we constructed and validated a two-gene signature, which could be used to predict of the OS of NSCLC. This signature may act as a new reference for clinical treatment and is valuable for screening out high-risk patients who require intensive follow-up and personalized intervention.

## Supplementary Information

**Additional file 1.**

## Data Availability

The datasets used in the current study are openly available in TCGA (https://cancergenome.nih.gov/) and GEO (https://www.ncbi.nlm.nih.gov/geo/) database and are available in the scienceDB repository (https://www.scidb.cn/s/AFfEva).

## Abbreviations

OS: Overall survival
NSCLC: Non-small cell lung cancer
LASSO: The least absolute shrinkage and selection operator
LUAD: Lung adenocarcinoma
TCGA: The cancer genome atlas
GEO: The gene expression omnibus
PTMs: Post-translational modifications
RSS: Risk scoring system
GSEA: Gene set enrichment analysis
OD: Optical density
DCA: Decision curve analysis
GO: Gene Ontology
