# Supplementary material for: Construction and validation of a two-gene signature based on SUMOylation regulatory genes in non-small cell lung cancer patients
Source: BMC Cancer. 2022 May 23;22:572. doi: 10.1186/s12885-022-09575-4 (PMC9125860; doi:10.1186/s12885-022-09575-4)
Supplement: Supplementary file 1 — Additional file 1. [file 12885_2022_9575_MOESM1_ESM.docx]

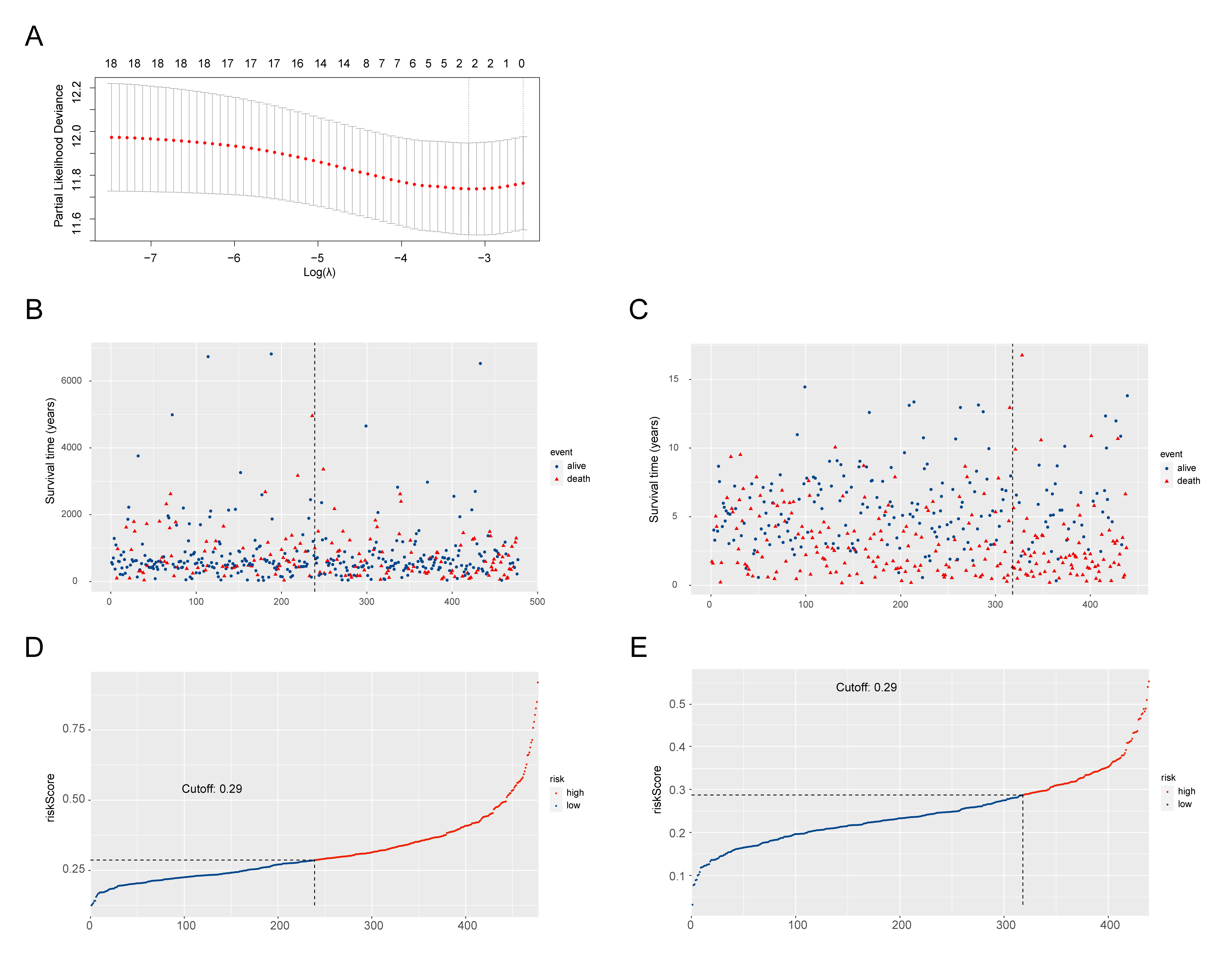


**Supplemental figure 1.** (A) The optimal penalty lambda (λ) selected by 10-fold cross-validation via minimum criteria. (B, C) Event distribution in TCGA training cohort and GSE68465 dataset, respectively. (D, E) Risk score distribution of risk genes in TCGA training cohort and GSE68465 dataset, respectively.


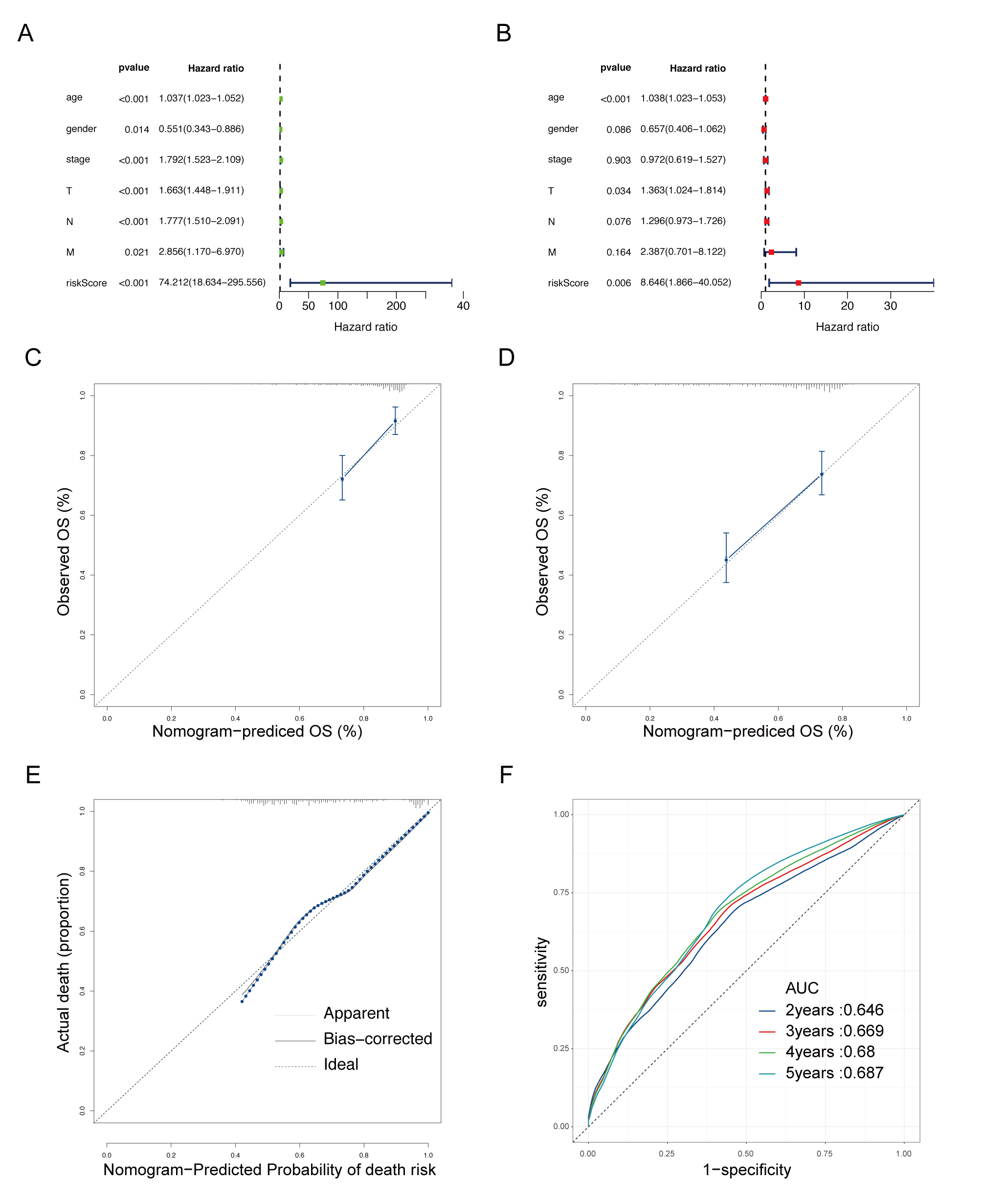


**Supplemental figure 2.** Validation of the risk signature and nomogram in the GEO dataset. (A, B) The univariate and multivariate Cox model between risk score and clinicopathological characteristics in the GSE30219 dataset. (C-E) The predicted 1-year, 3-year, and the calibration curves of NSCLC patients in the GSE30219 dataset are based on the nomogram. (F) The ROC curve analysis displayed the prediction accuracy of the prognostic signature in the GSE30219 dataset.


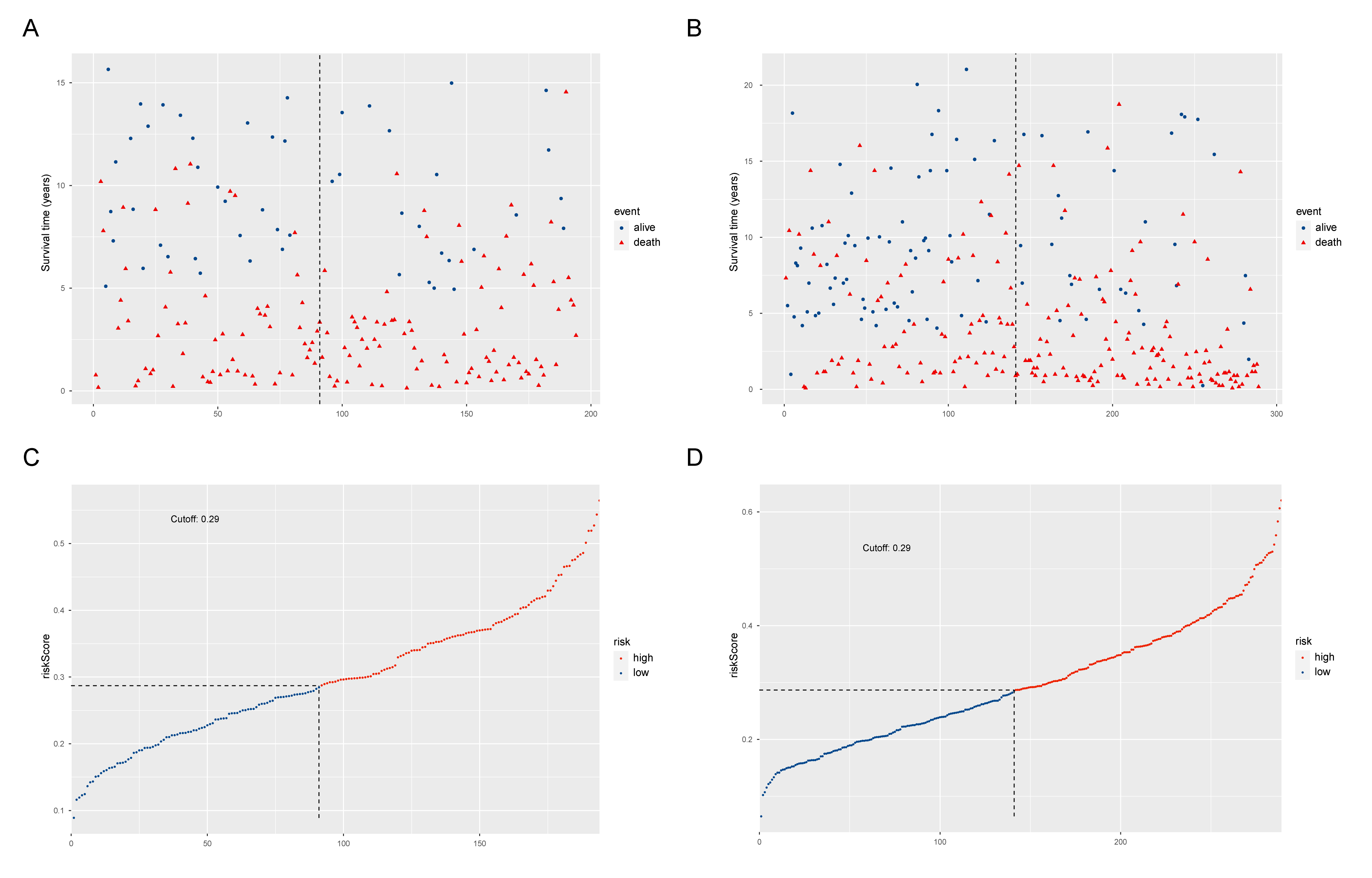


**Supplemental figure 3**. (A, B) The event distribution of *SAE1* and *UBA2* in NSCLC validation cohorts, respectively. (C, D) Risk score distribution of *SAE1* and *UBA2* in NSCLC validation cohorts, respectively.


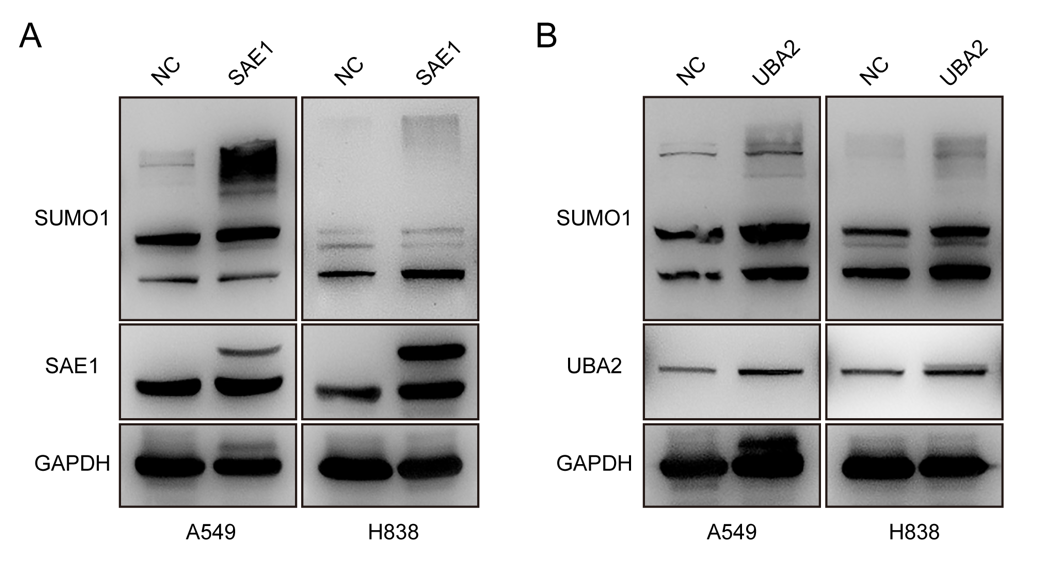


**Supplemental figure 4**. The transfect efficiency of risk genes plasmid in NSCLC cells. (A) The transfect efficiency of *SAE1* plasmid and the expression of SUMO1 after *SAE1* overexpression detected by western blot in NSCLC cell lines. (B) The transfect efficiency of *UBA2* plasmid and the expression of SUMO1 after *UBA2* overexpression detected by western blot in NSCLC cell lines.

**Table 1.** **Clinicopathological characteristics of patients in TCGA training cohort.**

| **Clinical**  **characteristics** | **Alive (n=325)** | **Death (n=157)** | **Total (n=482)** |
| --- | --- | --- | --- |
| **Gender** |  |  |  |
| Male | 149 (46 %) | 77 (49 %) | 226 (47 %) |
| Female | 176 (54 %) | 80 (51 %) | 256 (53 %) |
| **Age (years)** |  |  |  |
| Mean (SD) | 65 (± 9.7) | 66 (± 11) | 65 (± 10) |
| **Stage** |  |  |  |
| I | 206 (63 %) | 54 (34 %) | 260 (54 %) |
| II | 73 (22 %) | 47 (30 %) | 120 (25 %) |
| III | 36 (11 %) | 44 (28 %) | 80 (17 %) |
| IV | 10 (3 %) | 12 (8 %) | 22 (5 %) |
| **T** |  |  |  |
| T1 | 125 (38 %) | 41 (26 %) | 166 (34 %) |
| T2 | 168 (52 %) | 86 (55 %) | 254 (53 %) |
| T3 | 25 (8 %) | 19 (12 %) | 44 (9 %) |
| T4 | 7 (2 %) | 11 (7 %) | 18 (4 %) |
| **N** |  |  |  |
| N0 | 244 (75 %) | 73 (46 %) | 317 (66 %) |
| N1 | 47 (14 %) | 46 (29 %) | 93 (19 %) |
| N2 | 32 (10 %) | 38 (24 %) | 70 (15 %) |
| N3 | 2 (1 %) | 0 (0 %) | 2 (0 %) |
| **M** |  |  |  |
| M0 | 215 (66 %) | 109 (69 %) | 324 (67 %) |
| M1 | 10 (3 %) | 12 (8 %) | 22 (5 %) |
| Mx | 100 (31 %) | 36 (23 %) | 136 (28 %) |

**Table 2. Clinicopathological characteristics of patients in GSE68465 validation cohort.**

| **Clinical**  **characteristics** | **Alive (n=204)** | **Death (n=232)** | **Total (n=436)** |
| --- | --- | --- | --- |
| **Gender** |  |  |  |
| Male | 86 (42 %) | 133 (57 %) | 219 (50 %) |
| Female | 118 (58 %) | 99 (43 %) | 217 (50 %) |
| **Age (years)** |  |  |  |
| Mean (SD) | 63 (± 11) | 66 (± 9.4) | 64 (± 10) |
| **T** |  |  |  |
| T1 | 84 (41 %) | 65 (28 %) | 149 (34 %) |
| T2 | 114 (56 %) | 134 (58 %) | 248 (57 %) |
| T3 | 5 (2 %) | 23 (10 %) | 28 (6 %) |
| T4 | 1 (0 %) | 10 (4 %) | 11 (3 %) |
| **N** |  |  |  |
| N0 | 170 (83 %) | 127 (55 %) | 297 (68 %) |
| N1 | 27 (13 %) | 60 (26 %) | 87 (20 %) |
| N2 | 7 (3 %) | 45 (19 %) | 52 (12 %) |
